# Comparison of Three PCR-based Methods for Simplicity and Cost Effectiveness Identification of Cutaneous Leishmaniasis Due to *Leishmania tropica*

**Published:** 2017

**Authors:** Mohammad Ali MOHAMMADI, Mehdi BAMOROVAT, Majid FASIHI HARANDI, Tayyebeh KARIMI, Iraj SHARIFI, Mohammad Reza AFLATOONIAN

**Affiliations:** 1. Research Center for Hydatid Disease in Iran, Kerman University of Medical Sciences, Kerman, Iran; 2. Leishmaniasis Research Center, Kerman University of Medical Sciences, Kerman, Iran; 3. Dept. of Medical Parasitology, School of Medicine, Kerman University of Medical Sciences, Kerman, Iran; 4. Research Center for Tropical and Infectious Diseases, Kerman University of Medical Sciences, Kerman, Iran

**Keywords:** Molecular identification, Cutaneous leishmaniasis, PCR, Iran

## Abstract

**Background:**

To compare three molecular methods, PCR-RFLP for internal transcribed spacer, PCR sequencing and high resolution melting analysis shown reliable sensitivity and specificity for detecting *Leishmania tropica* as a model for cutaneous leishmaniasis (CL) as the perspective overview for scientific and economic approaches.

**Methods:**

This study was carried out between 2015 and 2016 in Leishmaniasis Research Center in Kerman University of Medical Sciences, Kerman, Iran. The positives smears (n=50) were obtained from patients referred from the health clinics in a major anthroponotic CL (ACL) focus, southeastern Iran. Only smear preparations with the same grade were selected according to the method described by the WHO for future PCR assays.

**Results:**

All three molecular methods had capability to identify positive samples at species level with the same specificity and sensitivity. However, these techniques were different in simplicity, consuming time, and cost effectiveness. Although additional enzymatic process in PCR-RFLP provided good resolution to find *Leishmania* species but this would cause time and cost increases.

**Conclusion:**

HRM (high resolution melting) is a relatively new technique that allows direct characterization of PCR amplicons in a closed system with more simplicity, cost effectiveness and time-consuming compared with other PCR-based assays for epidemiological or clinical identification purposes.

## Introduction

Leishmaniases are infectious diseases widespread in the world with high clinical and epidemiological diversity caused by many species of the genus *Leishmania* (1). This disease is still an important public health problem in 98 countries and territories, affecting both rural and urban regions worldwide. There are an estimated 0.2–0.4 million new cases of visceral leishmaniasis (VL) and 0.7–1.2 million new cases of cutaneous leishmaniasis (CL) annually (2). Over 21 *Leishmania* species have been observed to cause human infection (3, 4). Different clinical and epidemiological forms of anthroponotic CL (ACL) and zoonotic CL (ZCL) have been reported from several areas of Iran (5–8). Diagnosis and characterization of *Leishmania* species at species level are important for prognosis, epidemiological and therapeutic purposes (9).

Detection of amastigotes in lesions has relied upon direct microscopy of smear preparation by Giemsa staining, culturing and biopsies. A sensitive and specific method allowing the definitive diagnosis of parasites is extremely essential particularly when there are low numbers of amastigotes in the lesion.

Molecular techniques are increasingly employed for diagnostic and epidemiological purposes in order to confirm *Leishmania* infection and to characterize the parasites at the species or genotype level in humans and other hosts. Comparison of diagnostic methods in CL over the past years emphasized the use of PCR-based techniques approaching a ‘gold standard’ status offering considerable advantages in the collection and transport of specimens and DNA extraction procedures. These methods are more efficient in individuals and for field-based protocols because of their high specificity and sensitivity (10–13).

Various PCR-based molecular techniques have been employed such as conventional and nested-PCR, randomly amplified polymorphic DNA (RAPD), PCR-restriction fragment length polymorphism (RFLP), and in recent years high-resolution melting (HRM) curve analysis, which are powerful techniques for identification and phylogenetic studies in *Leishmania* (13–18). Accurate and sensitive diagnoses of *Leishmania* species/strains are essential for adequate treatment and appropriate public health control measures. Regarding PCR-based assays, they were found to be rapid, sensitive and discriminative at species or even strain level. However, the diagnosis of leishmaniasis remains a scientific challenge for researchers and clinical diagnostic laboratory according to distinct indicators such as sensitivity, specificity, cost effectiveness and time-consuming for each test (13).

While a number of studies have examined the accuracy of different PCR assays, few studies have evaluated simplicity, cost and time taking parameters. Therefore, the aim of this study was to compare three PCR methods shown reliable sensitivity and specificity for detecting *Leishmania tropica* as a model for CL based on literature reviews as the perspective overview for scientific and economic approaches.

## Materials and Methods

### Ethical consideration

This project (92/448 and 91/350) were approved by the Ethical Committees of the Leishmaniasis Research Center and Kerman University of Medical Sciences. Written consent of the cases was obtained. Patients were referred for treatments and possible flow-up examination.

### Sampling

The specimens were prepared from the patient referred from the health clinics in a major ACL focus in Kerman Province, southeastern Iran during 2015–2016. Tissue scraping was obtained from the periphery of each lesion by a scalpel and blade. The sample was smeared on a glass slide, fixed with methanol, stained by standard Gimsa and observed under a light microscope for the presence of amastigotes (Leishman bodies). Positive smear preparations were the source for further PCR assays. However, only highly positive slide preparations with the same grade (load of amastigotes) were selected for comparison purposes among the three methods (19).

### Evaluation performance of methods

The time taken for each test was calculated from the extraction of DNA until the interpretation of the result. Cost for each method was measured based on average commercial kit commonly used in research and diagnosis for DNA isolation and purification (DNeasy Blood & Tissue Kit, Qiagen-Germany), PCR kit (Taq PCR Master Mix Kit, QIAGEN-Germany), restriction enzyme Hae III (Thermo Fisher Scientific-US) and Type-it HRM PCR Kit (QIAGEN-Germany) for HRM.

DNA was extracted from the direct smear preparations following the manufacturer’s instructions. The DNA was stored at −20 °C until being used. Regarding the efficacy of the assay, it depends on the target selected for amplification (conserved or variable target region) and the number of the target copies(13), so the amplification region was selected for each PCR-based method using specific primers according to previous studies. Sequences of primers and amplification are summarized in Table 1.

**Table 1:** Selected amplification region for each PCR-based method based on previous studies

**PCR-based method**	**Target**	**PCR product size (bp)**	**Sensitivity %**	**Specificity %**	**References**
**PCR-RFLP**	Internal transcribed spacer 1 (ITS1)	300–350	92–100	100	[15,19,20]
**PCR-Sequencing**	7SL RNA	184–187	100	100	[21,22]
**HRM**	7SL RNA	119	100	100	[23]

For PCR-RFLP the reaction was carried out with the master mix in 25 μl of total reaction and based on the following conditions: initial denaturation at 94 °C for 3 min, followed by 38 cycles including denaturation at 94 °C for 30 sec, annealing at 53 °C for 35 sec, extension at 72 °C for 45 sec, and final extension at 72 °C for 5 min. At the end, 5 μl of the reaction mix was analyzed by 1.5% agarose gel electrophoresis to confirm PCR-specific amplification. Three samples of *L. trpoica, L. infantum,* and *L. major* species were amplified and loaded separately on gel as positive controls. Ten μl of 300–350 bp of amplicons were digested with Hae III enzyme, according to the manufacturer’s instructions. The restriction fragments were analyzed on 3% agarose gels by electrophoresis and visualized by UV light after being stained with ethidium bromide. The 100 bp DNA size marker mix was used as the DNA molecular ladder.

A PCR was used to high resolution melting curve analysis of the 7SL RNA performed as follows: template (2 μl, 20 ng/reaction) were added to 10 μl of 2× HRM kit, primers (0.5 μM [each] final concentration), and ultra-pure PCR-grade water, final volume, 20 μl/PCR. Amplification conditions were as follows: 95 °C denaturation for 15 min, followed by 40 cycles of denaturation at 95 °C for 5 sec; 55 °C annealing for 10 sec; and 20 sec at 72 °C for extension. Data were collected at the end of extension segment. HRM ramping was carried out at 0.1 °C/sec from 75 °C to 95 °C. HRM PCR and analysis were performed using a Rotor-Gene Q real-time thermal analyzer Software (Qiagen). Normalized melt window, 86 °C to 92 °C, was used in analyzing HRM curves.

The PCR processing and sequencing for 7SL RNA amplicons were prepared by the specific primers previously described. The products of positive samples were purified and sequenced by Macrogen Company (South Korea). Sequence similarity searching for each single sample was performed using the NCBI BLAST program. For phylogenetic study, analyses of multiple sequence alignments were done using the software BioEdit ver.6 (20) and MEGA ver.6 (21). The Kimura 2 parameter model was used to build the distance matrix and the tree was inferred using the Maximum Likelihood approach. Bootstrap resampling 1000 was done and a bootstrap consensus tree was produced. Nucleotide sequence data were reported in this article has been submitted to the GenBank database with accession numbers KT279392 - KT279397.

## Results

**Fig. 1:**
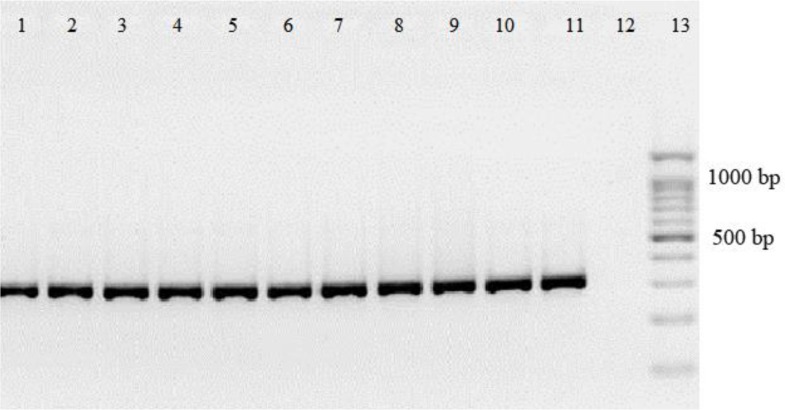
The PCR-based detection of *Leishmania tropica* amastigotes in smear representative from Kerman district, southeast of Iran. The amplified 300–350 bp product, from the positive control sample *L. trpoica* (lane 1), positive test samples (lanes 2–11) and none template control (lane 12). A 100 bp DNA size marker (Lane 13)

**Fig. 2:**
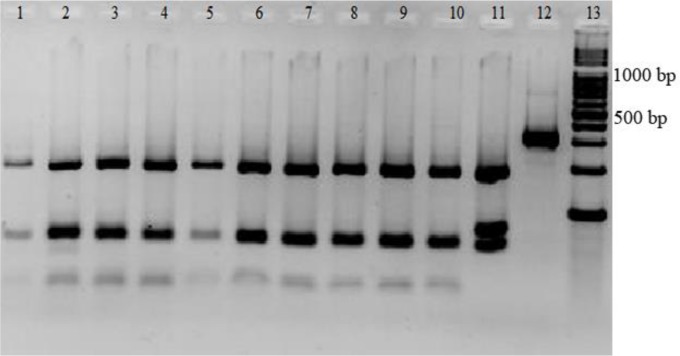
Three-percentage agarose gel electrophoresis of ITS1-PCR restriction fragments. Positive test samples (lanes 1–10), *L. major* (lane 11), and uncut PCR product (lane 12). A 100 bp DNA-size marker (Lane 13)

PCR-RFLP with specific primers for ITS1 resulted in the amplification of the *Leishmania* positive samples, giving 300 to 350 bp amplification bands (Fig. 1). Restriction of the ITS1-PCR products with the restriction enzyme Hae III generated patterns with two bands of 185 and 58 bp for *L. trpoica* (Fig. 2).

**Fig. 3:**
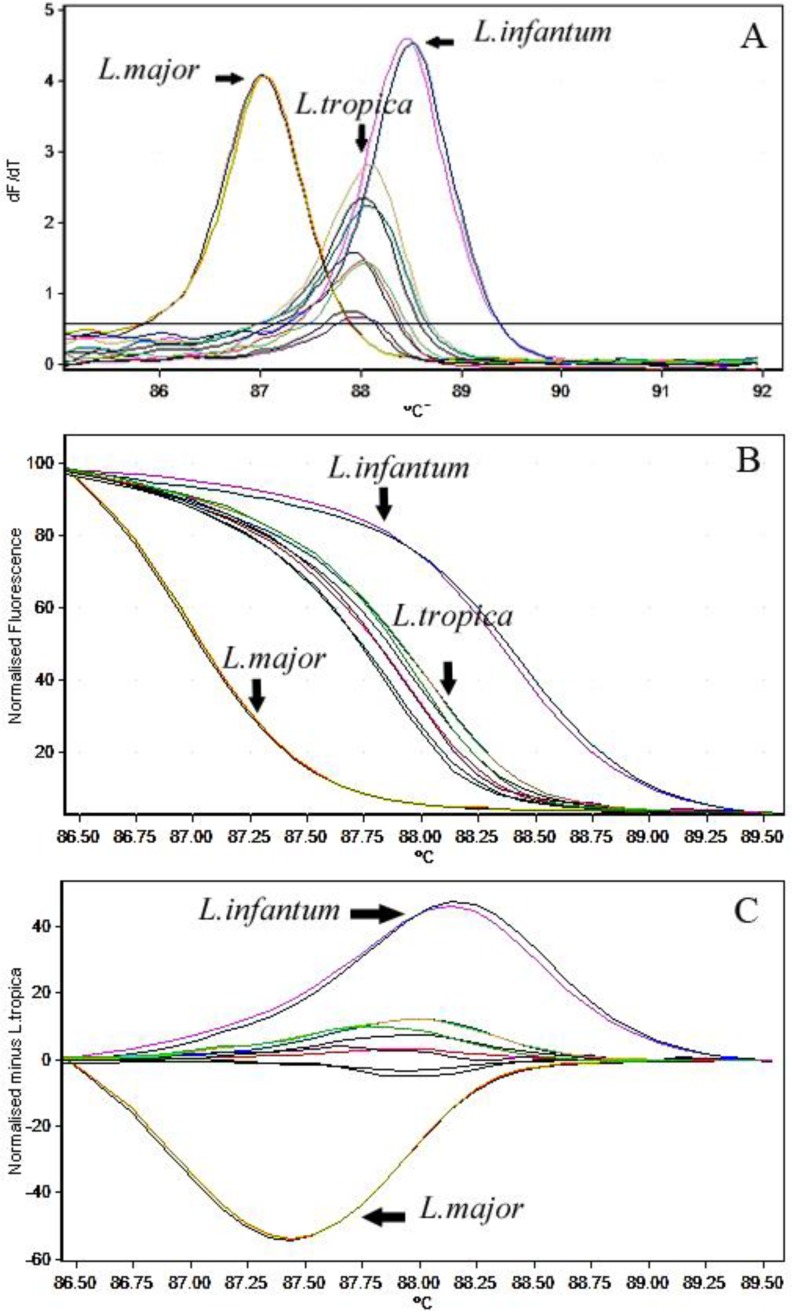
Comparison of normalized melting curve analysis. (A) Normalized high resolution melting curves, (B) Normalized high-resolution melting curves minus *L. tropica,* and (C) of the 7SL PCR amplicon. All three species (*L. tropica, L. major* and *L. infantum*) have been isolated in separate clusters

The discrimination between controls and positive samples in HRM curve is shown in Fig. 3. The average melting point (*T*_m_) ± standard deviation (SD) for 7SL RNA amplicons was calculated as follows: *L. major*, 87.03 ± 0.03 °C; *L. trpoica,* 88.09 ± 0.35 °C and *L. infantum*, 88.86 ± 0.02 °C.

**Fig. 4:**
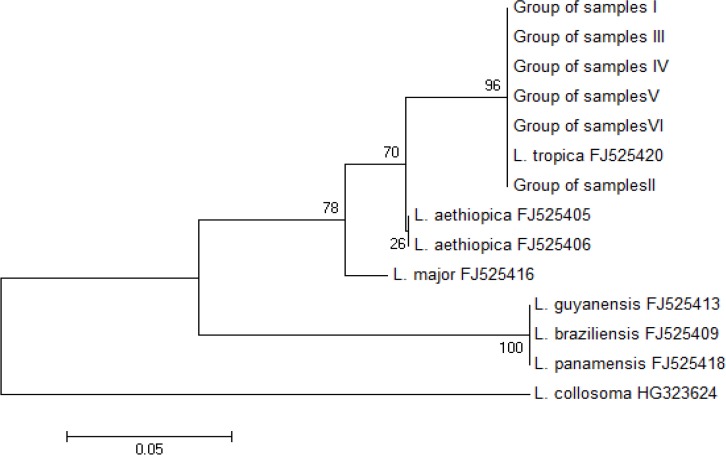
A phylogenetic tree rooted by out-group (*L. collosoma*), inferred from partial 7SL RNA nucleotide sequences using the Neighbor-Joining method after Kimura2 correction. Scale bar indicates the proportion of sites changing along each branch. All group samples were classified in the same clade with *L. trpoica* FJ525420

The partial amplification of 7SL RNA sequences for all positive samples clustered into five groups based on nucleotide polymorphism. However, all sequences were classified in the same clade with *L. trpoica* FJ525420. The clustering of a representative selection of the 7SL RNA sequences is depicted in Fig. 4.

Both sensitivity and specificity were calculated for each method according to the relevant articles (10, 15–19). All prices for services and commercial kits were calculated according to the basic tariff in 2015 online shopping web-sites. Measurements of each method outcome indicators are summarized in Table 2.

**Table 2:** Comparison of the three PCR-based methods by time-consumingness, cost and validity of the tests

**PCR-based method**	**Time-consumingness**	**Cost per test**	**Sensitivity %**	**Specificity %**
**PCR-RFLP**	7.5h*	$;5.72	100	100
**PCR-Sequencing**	3–7 days	$11.2	100	100
**HRM**	1.5h	$4.46	100	100

h= Hour

## Discussion

In this study, we tried to evaluate the ability of three PCR-based methods to discriminate between *Leishmania* species in terms of simplicity and cost-benefit. While the sensitivity and specificity of PCR assays for detecting *Leishmania* parasites have frequently been compared to those of conventional parasitological techniques, rarely have these parameters been evaluated in parallel for several PCR-based assays (11,13).

Researchers, clinical laboratories, and insurers choose laboratory test requirements to maximize the relationship between sensitivity, specificity, simplicity and cost-benefits (22–25). Molecular techniques could play an important role in detection and the monitoring of therapy in humans and animals leishmaniasis (26, 27). Based on our study, the three molecular methods confirmed that all 50 positive samples belong to *L. tropica* species. PCR with different specific *Leishmania* primers is widely used in clinical diagnosis, because o f the high specificity and sensitivity of the techniques to detect *Leishmania* amastigotes (5,11, 28). Variable results have been reported by several studies evaluating PCR using different target sequences in different host tissues. These results have been mostly obtained from infected hosts and they may vary depending on the PCR protocol employed (13). Moreover, additional endonuclease enzymatic process provides good resolution to find *Leshmania* species (16). The ability of PCR-RFLP for ITS1 partial amplification region to discriminate *Leishmania* species was reported, which is consistent with our results. However, additional process is costly and increases the likelihood of contamination and error in the results.

On the other hand, benefits of using sequencing as great tools to distinguish *Leishmania* species and nucleotide changes in phylogenic study was previously reported (29,30). Based on the sequencing results we are able to study the nucleotide composition and evaluate nucleotide changes in closely related species. Although some nucleotide variations were observed, all our positive samples belonged to *L. tropica* species and these differences were not significantly meaningful. The use of this technique needs more time for analysis and somewhat more expensive.

New molecular tools based on fluorescence detection such as HRM curve analysis is a relatively new technique that allows direct characterization of PCR amplicons in a closed system(16). This makes the ability to measure changes in the fluorescence intensity of a DNA intercalating dye during dissociation from double-stranded DNA to single-stranded DNA and can differentiate between nucleotide compositions. So far, HRM has not been extensively used in clinical diagnosis with leishmaniasis, but currently, numerous articles have pointed to the diagnostic capabilities of this technique. Rapid diagnosis of the Old World leishmaniasis by HRM analysis of the 7SL RNA gene fully confirmed by our findings (31). Another real-time ITS1-PCR based method was reported for the diagnosis and species identification of the Old World *Leishmmania* parasites (32). The proposed method had sufficient sensitivity for diagnostic purposes directly from clinical samples, as well as epidemiological studies, reservoir host investigations and vector surveys. Besides, for epidemiological study, the HRM method was used to characterize *Leshmania* species in natural *Leishmania* infection of *Phlebotomus sergenti* in south-eastern Iran, for the first time (33).

## Conclusion

All three molecular methods have capability to identify positive samples at species level. However, these techniques are different in consuming time and cost effectiveness. PCR-RFLP can be performed from beginning to the end in one diagnostic laboratory but additional enzymatic process to distinguish *Leishmania* species needs to spend more time for each test. Despite the immense benefits, taking more time and money in PCR-sequencing technique compared with other methods, sequencing as a clinical diagnosis is not the first option. The primary equipment performing HRM analysis is fairly expensive and sophisticated, which is the main limitation. Although it represents the priority benefits for its simplicity, cost effectiveness and rapidity compared with conventional PCR assays as justified for large-scale epidemiological studies or clinical identifications.
